# Case report: A case of novel treatment for retrograde cricopharyngeal dysfunction

**DOI:** 10.3389/fneur.2022.1005655

**Published:** 2022-12-21

**Authors:** Mengshu Xie, Hongmei Wen, Zulin Dou

**Affiliations:** Department of Rehabilitation Medicine, The Third Affiliated Hospital, Sun Yat-sen University, Guangzhou, Guangdong, China

**Keywords:** inability to belch, retrograde cricopharyngeal dysfunction, botulinum toxin, flatulence, electromyography

## Abstract

Retrograde cricopharyngeal dysfunction (R-CPD) is a recently described disorder characterized by an inability to belch, excessive flatulence, unpleasant gurgling noises, and discomfort in the lower neck, chest, and abdomen. Herein, we describe a case of R-CPD in a 19-year-old man. The patient suffered from flatulence and was unable to belch since birth; auxiliary examination of his digestive system was normal. He was diagnosed with R-CPD based on clinical manifestations and laboratory results. He received an injection of botulinum toxin to the cricopharyngeal muscle under ultrasound, catheter balloon, and electromyographic guidance. His symptoms completely resolved 1 week after the injection.

## 1. Introduction

Retrograde cricopharyngeal dysfunction (R-CPD) was first described in 2019 by Bastian et al. and it is characterized by an inability to belch, excessive flatulence, unpleasant gurgling noises, and discomfort in the lower neck, chest, and abdomen (1). The results of auxiliary examinations will be normal. Bastian and Smithson (1) and Bastian and Hoesli (2) reported the usage of botulinum toxin (BTX) injection for the treatment of R-CPD using two guidance methods: laryngoscopic guidance under general anesthesia and piercing of the cricothyroid membrane and traversing the lumen, posterior cricoid plate, and posterior cricoarytenoid muscles to reach the cricopharyngeal muscle with electromyographic guidance under topical anesthesia. These two methods have a relatively high need for equipment and technology. BTX injection into the cricopharyngeal muscle is still off-label. In addition to BTX injection into the cricopharyngeal muscle, partial cricopharyngeal myotomy can be used as another treatment option for patients who do not respond well after multiple injections (3). We performed and report a novel method of percutaneous BTX injection into the cricopharyngeal muscle with ultrasound, catheter balloon, and electromyographic guidance for the treatment of R-CPD without anesthesia.

## 2. Case description

A 19-year-old man suffered from flatulence since birth, which aggravated when he cried and even caused vomiting. He was unaware that people could belch to release flatulence until college. It was then that he realized his differences from other people and began to visit the hospital. He was 180-cm tall but weighed only 54 kg. He had flatulence and was unable to belch, but he did not have clinical dysphagia. Laboratory examination results, including routine blood examination, biochemical index, and rheumatism indicators, were normal. Auxiliary examinations such as gastroscopy (performed twice), abdominal ultrasound, abdominal enhanced computed tomography (CT), and esophageal barium meal examination revealed only chronic superficial gastritis. Medicines such as proton-pump inhibitors and anxiolytic agents were ineffective.

**Figure 1:**
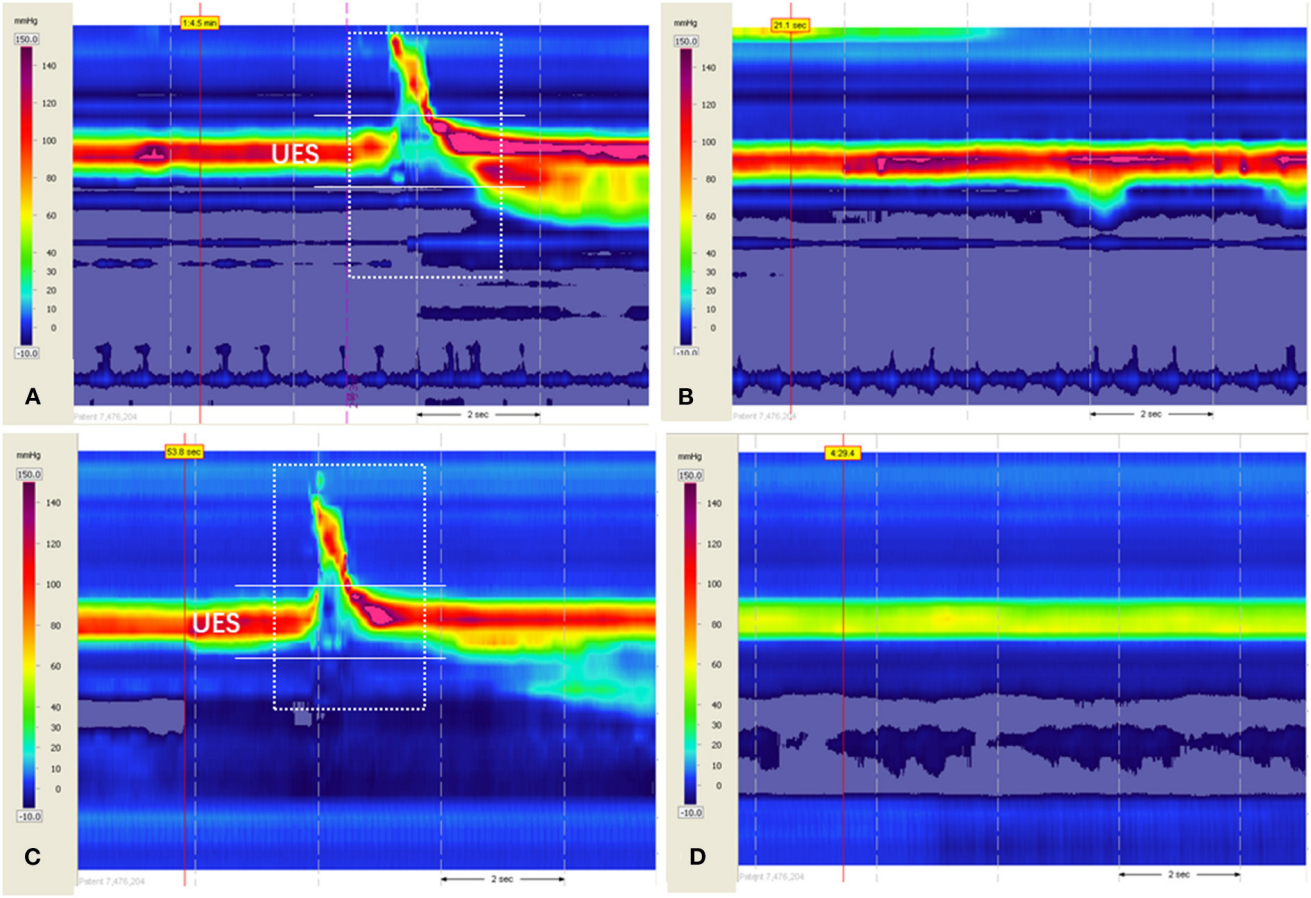
A space–time diagram of high-resolution manometry before and after treatment. The *x*-axis represents time (dotted arrow indicates 2 s), and the *y*-axis represents the distance from the nostril. Each pressure was assigned a color (legend, left). **(A)** When swallowing 3 ml of thick liquid before treatment on April 6, 2021, the UES resting pressure and UES residual pressure increased, while the coordination of pharyngeal contraction and UES relaxation was normal. **(B)** Resting pressure in the resting state before treatment on April 6, 2021. **(C)** When swallowing 3 ml of thick liquid after treatment on July 22, 2021, the UES resting pressure and UES residual pressure dropped to normal. **(D)** The resting pressure in the resting state after treatment on July 22, 2021, was a more straightforward indication of improvement. The white arrow indicates the UES residual pressure. The dotted box indicates swallowing. UES, upper esophageal sphincter.

**Figure 2:**
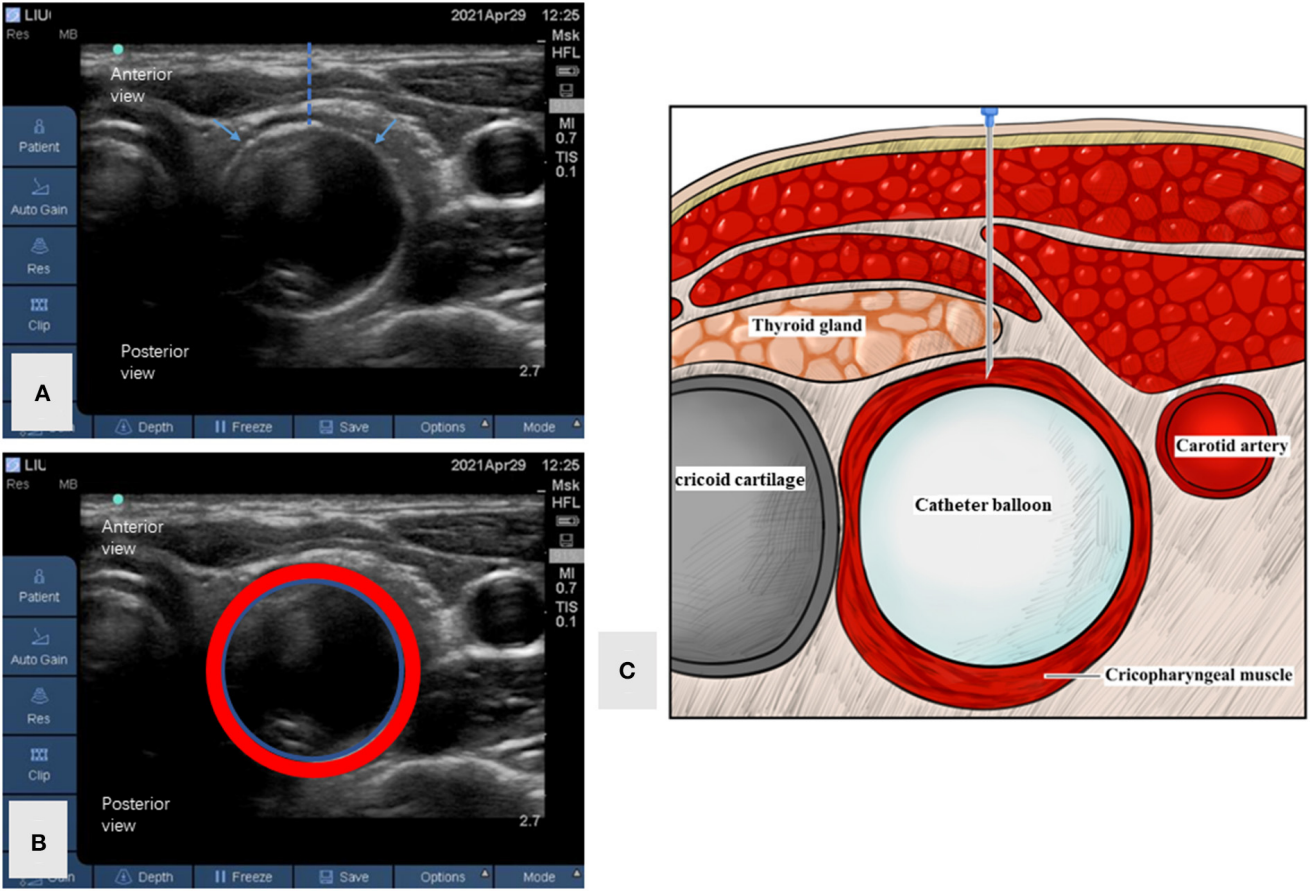
Ultrasound imaging of the cricopharyngeal muscle during the operation. **(A)** The ultrasound imaging of the cricopharyngeal muscle during the operation. The blue arrow shows the cricopharyngeal muscle under catheter balloon guidance. The dotted line indicates the expected injection path. **(B)** The ultrasound imaging of the cricopharyngeal muscle and catheter balloon with margins drawn. The red circle shows the cricopharyngeal muscle. The blue circle shows the catheter balloon. **(C)** The cartoon of cricopharyngeal muscle and adjacent structures.

The patient visited our clinic for assistance. Additional auxiliary examinations were performed. Flexible endoscopic evaluation of swallowing (FEES) and videofluoroscopic swallowing study (VFSS) ruled out structural abnormalities in the oral cavity, pharynx, and larynx. FEES revealed only trace residues in the vallecula epiglottica. VFSS showed a normal opening of the cricopharyngeal muscle and trace residues in the vallecula epiglottica when swallowing. The patient also underwent high-resolution manometry (HRM), which revealed increased upper esophageal sphincter (UES) residual pressure (22.6 mmHg, normal reference value < 12 mmHg) and normal hypopharyngeal peak pressure, velopharyngeal peak pressure, and coordination of swallowing. Although the UES resting pressure was within the normal range (76.6 mmHg; normal reference range: 34–104 mmHg), the space–time diagram was quite red, indicating high pressure (Figures 1A,B). Based on his symptoms and examination findings, we diagnosed him with R-CPD. Once diagnosed, BTX injection (BOTOX, 50 U) with ultrasound, catheter balloon, and electromyographic guidance was performed after obtaining informed consent from the patient and his parents on April 29, 2021 (Figure 2). BTX injection with combined guidance has been skillfully used in clinical practice (4, 5). A catheter balloon was inserted into the esophagus through the nasal cavity, and 5 ml of water was injected into the balloon. When the balloon is pulled up gently, it can be stuck at the cricopharyngeal muscle. Under ultrasound guidance, the cricopharyngeal muscle with the balloon can be observed clearly. An electromyography needle was then inserted under ultrasound guidance. A change in the electromyography signal was heard when the needle passes through the thyroid and into the cricopharyngeal muscle. BTX (50 U) was diluted with normal saline (0.5 ml) and injected into the cricopharyngeal muscles. The entire operation lasted ~20 min.

When followed up by telephone 1 week after the injection, the patient reported that he could belch by turning his head to the right, and that his abdominal distension improved significantly. Re-examination of FEES and VFSS on July 22, 2021, was normal, and the UES residual pressure dropped to normal levels during swallowing (5.5 mmHg), as did the UES resting pressure (Figures 1C,D). His symptoms did not recur for a year after the BTX injection. He did not gain much weight during this period. No adverse events such as transient dysphagia or alterations of the voice were reported after the treatment. Written informed consent was obtained from the individual for the publication of any potentially identifiable images or data included in this article.

## 3. Discussion

The cricopharyngeal muscle, the main component of the UES, is a high-pressure zone that only relaxes transiently during swallowing to allow a bolus to enter the esophagus. It is a physical barrier that protects against reflux of food into the airways and prevents entry of air into the digestive tract (6, 7). CPD includes anterograde CPD (A-CPD) and R-CPD. A-CPD is usually characterized by symptoms such as dysphagia, regurgitation, and cough. A-CPD can result from a series of neurological diseases (8). R-CPD, which has an unknown etiology, is mainly manifested as four symptoms: inability to belch; excessive flatulence; loud, and therefore socially awkward gurgling noises; and daily discomfort in the lower neck, chest, and abdomen (2).

Under physical conditions, excessive air can stimulate the mechanoreceptor and cause transient UES relaxation, and the air can escape the UES (9). HRM of the patient in the present study revealed mildly increased UES residual pressure with normal pharyngeal contraction and coordination, which could provide enough pressure to relax the cricopharyngeal muscle with assistance of the pharyngeal constrictor and hyoid muscles when swallowing to allow the bolus to enter the esophagus, indicating unimpaired swallowing function. However, when flatulence occurs, the air in the stomach may find it difficult to break through the high UES pressure without the assistance of the pharyngeal constrictor and hyoid muscles, suggesting the inability of the cricopharyngeal muscle to perform retrograde relaxation and the inability to belch. Since no HRM results of these patients have been reported before, we hypothesized that the increased UES residual pressure could be one of the reasons for the symptoms of R-CPD. HRM is a potentially important examination for the diagnosis and evaluation of these patients. It was reported recently that high-resolution manometry and impedance monitoring (HRIM) can be valuable examinations for R-CPD patients. In patients with R-CPD, impaired UES relaxation without gas reflux could be detected after they drank carbonated water, while the UES relaxation was normal when swallowing (10, 11). This provided an objective examination basis for the clinical symptoms in these patients.

There have been many studies and clinical experiences in the treatment of A-CPD. BTX injection into the cricopharyngeal muscle can be an effective treatment method for A-CPD, which relies on the precise location of the cricopharyngeal muscle. At present, the commonly used guidance methods include CT, ultrasound, electromyography, and a combination of these methods. Generally, a single guidance method may not be accurate in positioning; therefore, most of the articles in the published literature reported a combination of several guidance methods. Bastian and Smithson (1) and Bastian and Hoesli (2) reported two methods of guidance: laryngoscopic guidance under general anesthesia, which has high requirements in terms of equipment and technology, and trans cricothyroid membrane puncture, which has high requirements for the operator in terms of skill and to precisely locate the puncture point. The proposed localization method is relatively simple and easy to implement. Based on our previous research and experience (4), we applied our treatment and location method for A-CPD to R-CPD and achieved a very good result. Although there are some reported adverse events in the literature, including transient dysphagia, hoarseness, and reflux after injection of BTX, this patient reported no side effects of the BTX treatment and procedure. This case indicates that BTX injection into the cricopharyngeal muscle with ultrasound, catheter balloon, and electromyographic guidance, as well the assistance of HRM, can be beneficial.

## Data availability statement

The raw data supporting the conclusions of this article will be made available by the authors, without undue reservation.

## Ethics statement

Written informed consent was obtained from the individual(s) for the publication of any potentially identifiable images or data included in this article.

## Author contributions

ZD and HW: patient diagnosis and treatment. MX: data collection and draft manuscript preparation. HW: critical revision of the manuscript. All authors reviewed and approved the final version of the manuscript.
